# Use of the CardioMEMS Device in Children and Patients with Congenital Heart Disease: A Literature Review

**DOI:** 10.3390/jcm13144234

**Published:** 2024-07-19

**Authors:** Enrico Piccinelli, Giorgia Grutter, Mara Pilati, Micol Rebonato, Silvia Teresa Scalera, Rachele Adorisio, Antonio Amodeo, Gessica Ingrasciotta, Erica Mencarelli, Lorenzo Galletti, Gianfranco Butera

**Affiliations:** 1 Bambino Gesù Children’s Hospital, IRCCS, 00165 Rome, Italygessica.ingrasciotta@opbg.net (G.I.);; 2 Department of Mechanical and Aerospace Engineering, Politecnico di Torino, 10129 Torino, Italy

**Keywords:** CardioMEMS, congenital heart disease, heart failure, heart transplantation

## Abstract

The CardioMEMS HF System (Abbott, Abbott Park, IL) is the first FDA- and CE-Mark-approved device for monitoring patients with heart failure, significantly reducing hospitalizations and improving the quality of life for NYHA class III non-congenital adult patients. This device, implanted percutaneously, allows the direct monitoring of pulmonary arterial pressure with the wireless transfer of pressure data to the clinician, who can adjust the therapy remotely. Limited experience exists regarding its use in patients with congenital heart disease (CHD). CardioMEMS device implantation is feasible and safe in selected adults and children with CHD. The potential of the device to reduce heart failure hospitalizations in this population is enormous, but further multi-center studies are needed to demonstrate its efficacy.

## 1. Introduction

The structural or functional impairment of ventricular filling or ejection of blood is a defining characteristic of heart failure (HF) [1]. The burden of HF is increasing, representing one of the leading causes of hospital admissions in developed countries [2,3]. Patients with pulmonary hypertension in the setting of HF have significantly higher morbidity and mortality [4]. In recent years, invasive and non-invasive telemonitoring technologies have emerged as tools to detect early signs of worsening heart failure [5,6].

The CardioMEMS HF System (Abbott, Abbott Park, IL) is the first FDA- and CE-Mark-approved device to monitor patients with heart failure, significantly reducing hospitalizations and improving quality of life for NYHA class III non-congenital adult patients [3,7,8,9]. This device, implanted percutaneously, allows the direct monitoring of pulmonary arterial pressure with the wireless transfer of pressure data to the clinician, who can modulate the therapy remotely. The potential of these implantable hemodynamic monitors (IHMs) is enormous, as detecting early signs of hemodynamic congestion may lead to more aggressive treatment to avoid new HF hospitalizations, even before the appearance of symptoms. The safety and efficacy of this IHM have been demonstrated in patients with normal hearts, but there are few reports about its use in patients with congenital heart disease (CHD). This review aims to investigate the state of the art and potential of this device in the growing population of adults living with complex CHD.

## 2. Procedure

**Figure 1:**
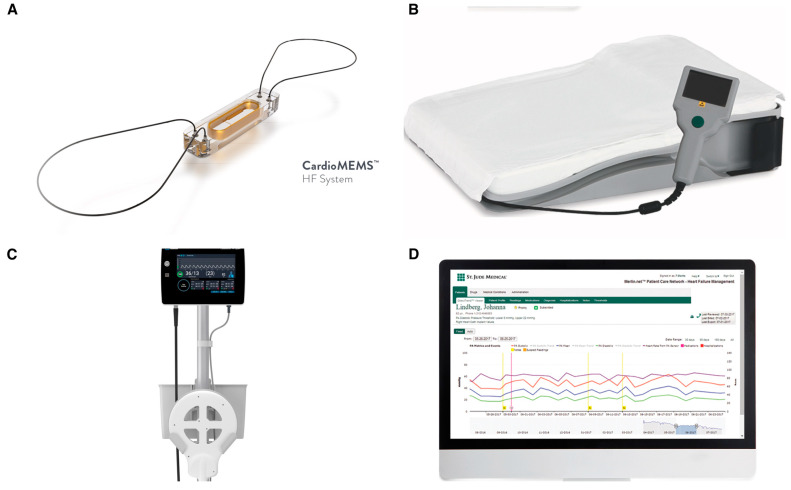
(**A**) The CardioMEMS device is made of a sensor measuring 3.5 mm in width, 2 mm in thickness and 15 mm in length. Two 10 mm diameter polytetrafluoroethylene-coated nitinol loops are attached to the two edges to maintain sensor apposition to the vessel wall after release. (**B**) An antenna is positioned in a pillow used by the patients to transmit data, while the “calibration wand” (**C**) is placed underneath the patient after device release for calibration. (**D**) Hemodynamic data are wirelessly transmitted to a secure website that serves as the patient database for pulmonary artery remote pressure monitoring.

The CardioMEMS device (Figure 1) is a radiopaque sensor, inserted via right heart catheterization, that wirelessly transmits hemodynamic data to a secure website that serves as the patient database so that PA monitoring information is always available to the physician. The sensor is inside a silicon-covered capsule and couples with an antenna positioned in a pillow that is used by the patients to transmit data. The central sensor is connected to two nitinol loops at each end of the sensor, which are designed to anchor the device within a pulmonary artery branch sized between 7 mm and 15 mm. The CardioMEMS is advanced over a 0.018″ wire usually through a 12 French long sheath in case of complex anatomies.

## 3. Possible Indications in Patients with Congenital Heart Disease

Patients with complex congenital heart disease (CHD) and advanced heart failure (NYHA class II-III) who have had prior hospitalizations for heart failure may benefit from CardioMEMS implantation. Different CHD sub-populations addressed in the literature include single-ventricle patients palliated with Fontan circulation and patients with d-transposition of the great arteries after atrial switch, congenitally corrected transposition of the great arteries and a borderline left ventricle [10,11,12,13,14].

The Fontan procedure is the final stage of three operations performed to palliate children born with single-ventricle physiology. In this operation, the systemic venous blood is diverted to the pulmonary arteries in the absence of a sub-pulmonic ventricle, resulting in a passive flow of blood through the pulmonary vasculature. This flow depends on a gradient between central venous pressure and a combination of pulmonary vascular resistance and systemic ventricle end-diastolic pressure. Even patients with well-functioning Fontan circulation have pressures in the system of 10–12 mmHg with chronic central venous congestion and low–normal cardiac output. Over time, this condition may lead to heart failure with worsening functional capacity and complications such as protein-losing enteropathy (PLE), plastic bronchitis (PB) and Fontan-associated liver disease (FALD) [15,16]. Higher pressures in the Fontan circulation increase the risk of these complications and mortality [17]; therefore, having a tool such as the CardioMEMS device that provides continuous pressure monitoring may lead to better management and outcomes in this population. This is especially important because these measurements reflect the daily-life hemodynamics of these patients, not being affected by common bias during catheterization such as fasting, sedation, aggressive diuretics therapy and, if necessary, mechanical ventilation. CardioMEMS implantation is possible both in Fontan with an extracardiac conduit and a lateral tunnel [10]. In the setting of Fontan circulation, the left pulmonary artery (LPA) is generally selected as the target vessel for device implantation, and the right pulmonary artery (RPA) is chosen mainly in the case of LPA hypoplasia or previous LPA stenting [10,11,12,18]. However, implanting the CardioMEMS distal to an LPA stent is possible by using a long sheath [11]. Moreover, CardioMEMs device implantation is feasible and safe also of in children with single-ventricle physiology palliated with Fontan. In a case series of six children with Fontan circulation, there were no device-related complications or caregiver concerns with regular follow-up [19]. Another experimental possibility can be suturing of the CardioMEMS pressure sensor into the extracardiac Fontan conduit during the standard Fontan operation, which was described by Rhodes al. on a child with pulmonary atresia, an intact ventricular septum, and a hypoplastic right ventricle with coronary artery sinusoids [20].

CardioMEMS implantation was also described in a child with a borderline left ventricle who underwent fetal aortic valvuloplasty and multiple surgeries, including the Ross procedure and prosthetic mitral valve placement. In this case, data collected from the CardioMEMS device and a simultaneous cardiopulmonary exercise test showed an increased mechanical mitral valve gradient and severe pulmonary hypertension, supporting the need for surgical mitral valve replacement [14].

## 4. Heart Failure Hospitalization Rate Reduction and Mortality

Despite the potential benefits of this technology, the results in terms of HF hospitalization reduction are controversial. Marshal et al. described a population of 17 NYHA II or III adult patients with Fontan circulation receiving a CardioMEMS, demonstrating that the IHM did not reduce HF hospitalizations, although there was low adherence to transmission [10]. Indeed, only in 46% of cases was there an IHM reading before HF hospitalization [10,12]. Bradley et al. noted that higher mean pulmonary artery pressures in adults with Fontan circulation were associated with an increased risk of heart failure-mediated events, defined as cardiovascular medication change, hospital admission, paracentesis and a change in heart transplant listing status in the 12 months after implantation (OR 1.17 [1.09, 1.25], *p* < 0.0001). They concluded that mean pulmonary artery pressures >24 mmHg or pressure changes >4 mmHg at CardioMEMS readings may be associated with more HF events [12]. In most of the studies, patients transmitted data two to three times a week. As expected, pressures at the time of hospitalization were higher than the baseline pressures. In many cases, HF hospitalization could be avoided by titrating diuretics [10].

Interestingly, IHM implantation is not changing the mortality rate in adult NYHA II or III patients with Fontan, despite confirmation of the association between higher pressures and mortality. Indeed, those who died during follow-up had significantly higher Fontan pressures than those who lived (median 19 vs. 14 mmHg; *p* < 0.001) [10]. These controversial results may be explained by a selection bias of patients with a failing Fontan circulation which is a totally different population in comparison to adults without CHD. In many centers, CardioMEMS implantation is the last therapeutic option in patients with multiple hospitalizations and already-optimized medical therapy after at least three cardiac surgeries, which may explain the mild efficacy of IMH in this setting, obtained in these preliminary studies. Moreover, heart failure and subsequent hospitalization may manifest in many ways in these patients with complications such as protein-losing enteropathy, plastic bronchitis and Fontan-associated liver disease.

## 5. Remote Monitoring

In most countries, the referral centers for complex congenital heart disease are far away and difficult to reach for patients, making frequent clinical evaluation socially and financially unsustainable [13]. The impact of IHM in this setting is crucial, reducing the number of physical outpatient examinations only when strictly needed. Moreover, the CardioMEMS device measurements reflect the normal activity hemodynamics, which can also change according to different altitudes. This is extremely important in Fontan circulation, where pressure difference of 1–2 mmHg may also play a significant role in terms of complications and long-term outcomes. Bhat et al. pointed out this issue by describing a symptomatic child with Fontan circulation and progressive liver fibrosis who had normal hemodynamics during catheterizations performed at a lower elevation and severely elevated Fontan pressures at CardioMEMS readings when returning home at a higher elevation (∼2100 m) [19].

## 6. Possible Adverse Events

### 6.1. Procedural Complication

**Figure 2:**
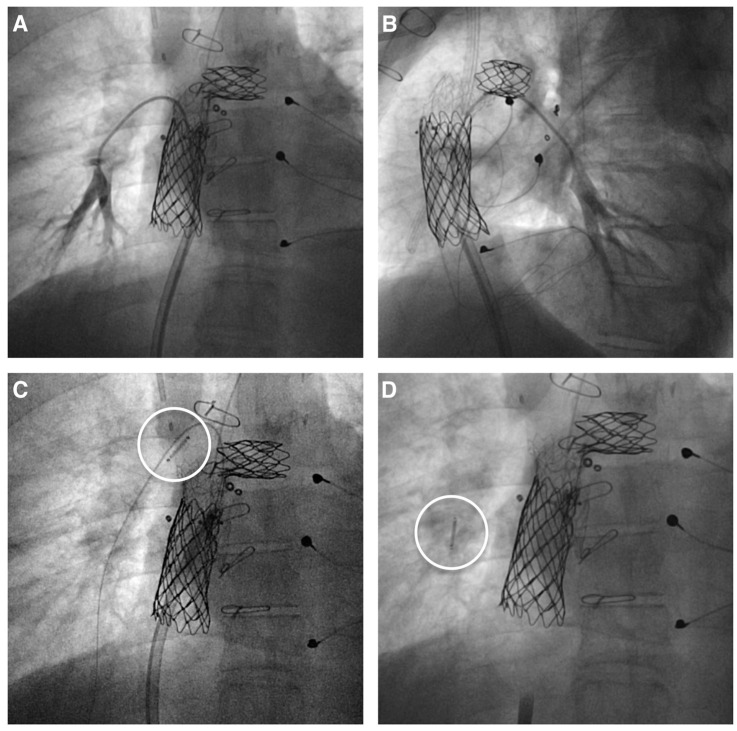
CardioMEMS implantation in Fontan patients. (**A**,**B**) A 7–8 mm right lower pulmonary artery branch is selected to accommodate the CardioMEMS device in a patient with a Fontan circulation who previously underwent conduit and left pulmonary artery stenting. (**C**) The device (white circle) is advanced over a 0.018″ wire, aided by a 12 French long sheath in the stented Fontan conduit. (**D**) The CardioMEMS device (white circle) in place after release in the selected pulmonary artery branch.

The CardioMEMS implantation can be slightly more challenging in patients with non-physiological anatomies after surgery such as Fontan circulation. However, with some technical modification, the procedure is feasible and safe. The use of a long sheath may help in the case of stents in the pulmonary arteries or in the Fontan conduit or lateral tunnel, avoiding the potential impingement of the device within the stent struts and facilitating the device’s advancement at the anastomosis level [10,11] (Figure 2). However, some procedural complications have been reported.

Device embolization has been described in patients after atrial switch operations, with device migration from the LPA into the distal right lower lobe PA branch the day following the procedure, even in the absence of significant pulmonary regurgitation [11]. Device shift, defined as a proximal repositioning of the device immediately upon release, in the absence of true migration to another vessel or chamber was also described in patients who underwent atrial switch operations. The authors described a proximal shift of the device by a mean of 5.9 ± 1.0 mm, mainly in patients where a larger target pulmonary vessel was selected (12.8 ± 1.0 mm vs. 8.6 ± 1.4 mm, *p* = 0.01) [11]. Therefore, the risk of device embolization or shift may be reduced, aiming for a target vessel diameter of 7–8 mm, as suggested by the company. Other described complications were stent distortion during long sheath advancement, pulmonary artery branch staining during hand angiography and minor vascular complications such as hematomas [10,11]. Some interference with the CardioMEMS device is also possible in the presence of a subcutaneous implantable loop recorder (ILR) in place in the left chest before IHM implantation. Procedural calibration and home transmitting readings may be difficult and resolved only with ILR removal [11]. No acute events of thromboembolism, vessel damage, valve injury or bleeding were described.

### 6.2. Long-Term Complications

Thromboembolic complications are rare (0.09%) after implantation of the CardioMEMS HF system according to postmarketing analysis [21]. However, pulmonary embolism has been described during follow-up in a Fontan patient, who developed a thrombus in the distal pulmonary artery branch of the PA, adjacent to the distal end of the CardioMEMS 54 months after device implantation. The adverse event seemed to be related to anticoagulation discontinuation due to gynecological bleeding and was successfully treated with a catheter-directed thrombolytic [10]. Another patient experienced a large thrombus within the Fontan conduit 3 years after CardioMEMS implantation, probably due to the presence of a chronic in-dwelling central line in the internal jugular vein for inotrope infusion, far away from the device located in the distal LPA [11]. The majority of other patients who underwent computed tomography angiography for other reasons did not show clinical or subclinical signs of device-related thrombosis [10,11,12].

CardioMEMS recalibration during follow-up was needed in six out of eight Fontan/dTGA-Mustard patients undergoing cardiac catheterization with pressure errors between 1 and 6 mmHg [10,11]. This is significantly higher than the 0.6% of patients requiring recalibration in CardioMEMS postmarketing analysis [21] but consistent with other reports of a mean difference of 4–6 mmHg at 2 months FU catheterization [22,23]. This is particularly important because a difference of 5–6 mmHg is relatively relevant in a biventricular circulation but certainly not acceptable in a Fontan circulation.

## 7. Therapy

Antiplatelet and/or anticoagulation therapy is a matter of debate in the congenital population after CardioMEMS implantation. The company suggests that patients not on chronic anticoagulant therapy should be placed on aspirin and clopidogrel daily for one month following sensor placement, followed by aspirin only to reduce the likelihood of thrombotic events. However, most of the Fontan patients who have a clinical indication for CardioMEMS implantation are already on anticoagulation therapy due to Fontan failure and other comorbidities. Nevertheless, there was a tendency to prescribe systemic anticoagulation after CardioMEMS implantation also in the patients not on anticoagulation therapy before, especially in the presence of a conduit fenestration. Most of these patients continued anticoagulation therapy indefinitely during follow-up, with a switch to antiplatelet therapy in a few cases [10,11]. Patients with dTGA/Mustard would benefit from antiplatelet therapy [11].

## 8. Future Directions

The CardioMEMS device may decrease the need for surveillance hemodynamic catheterizations and, with good patient adherence, reduce the number of HF hospitalizations. Patient education is crucial, with the full involvement of the families and caregivers, to optimize the potentiality of remote hemodynamic monitoring [16]. Many strategies may help improve adherence to the regular transmission of data. Patients and their caregivers should be educated about their disease and possibly involved in decision-making on their medication changes. Nurse practitioners may help in remote monitoring, contacting patients who miss CardioMEMS readings and querying about adherence to data transmission and medications. If well established in the ACHD population, its use can be expanded in HF pediatric populations such as those with failing Fontan or a failing graft after heart transplant or patients with ventricular assist devices. There is preliminary experience with CardioMEMS implantation in an adolescent affected by hypertrophic cardiomyopathy, advanced biventricular heart failure and severe pulmonary hypertension on SynCardia Total Artificial Heart (TAH) support. The IHM aided pulmonary hypertension management and medication modulation after TAH implantation until death due to sepsis [24]. Limiting factors such as delivery sheath size and adequate size branch pulmonary arteries can be overcome by the correct selection of patients.

## 9. Conclusions

Percutaneous implantation of the CardioMEMS device is feasible and safe in selected adults and children with CHD. The rate of procedural and long-term complications is low, thanks to technical modifications in the procedure and subsequent therapeutic management. The potential of the device in reducing HF hospitalization in this population is enormous, but further multi-centric studies are needed to demonstrate its efficacy. Uncertainty remains on the adherence to the regular transmission of hemodynamic data in the congenital population, which is, as expected, lower than the adult population without CHD. Moreover, the necessity of CardioMEMS recalibration over time in this setting needs clarification, as it is excessively high if compared with the postmarketing data available for the adult population without CHD. Finally, there is a notable lack of long-term data on the efficacy and safety of the CardioMEMS device in the CHD population. A significant gap in the current research exists, and further multi-center studies and trials are needed to establish robust medium- and long-term outcomes.

## 10. Limitations

There are several limitations of this literature review. First, there are only a few papers and case series addressing this topic, considering the novelty of the application of CardioMEMS in the congenital population. Second, most of these reports are single institutional retrospective experiences with a small sample size and lack of control groups, which limits the generalizability of the findings. Finally, it is difficult to provide robust outcomes on the efficacy of hospitalization reduction in this population due to the lack of regular transmission of hemodynamic data by patients in many reports.

## Data Availability

No new data were created or analyzed in this study.
